# Multi-omics approaches to studying gastrointestinal microbiome in the context of precision medicine and machine learning

**DOI:** 10.3389/fmolb.2023.1337373

**Published:** 2024-01-19

**Authors:** Jingyue Wu, Stephanie S. Singleton, Urnisha Bhuiyan, Lori Krammer, Raja Mazumder

**Affiliations:** ^1^ Department of Biochemistry and Molecular Medicine, School of Medicine and Health Sciences, The George Washington University, Washington, DC, United States; ^2^ Milken Institute School of Public Health, The George Washington University, Washington, DC, United States; ^3^ The McCormick Genomic and Proteomic Center, The George Washington University, Washington, DC, United States

**Keywords:** precision medicine, machine learning, gut microbiome, metagenomics, multi-omics, sequencing, biomarkers

## Abstract

The human gastrointestinal (gut) microbiome plays a critical role in maintaining host health and has been increasingly recognized as an important factor in precision medicine. High-throughput sequencing technologies have revolutionized -omics data generation, facilitating the characterization of the human gut microbiome with exceptional resolution. The analysis of various -omics data, including metatranscriptomics, metagenomics, glycomics, and metabolomics, holds potential for personalized therapies by revealing information about functional genes, microbial composition, glycans, and metabolites. This multi-omics approach has not only provided insights into the role of the gut microbiome in various diseases but has also facilitated the identification of microbial biomarkers for diagnosis, prognosis, and treatment. Machine learning algorithms have emerged as powerful tools for extracting meaningful insights from complex datasets, and more recently have been applied to metagenomics data via efficiently identifying microbial signatures, predicting disease states, and determining potential therapeutic targets. Despite these rapid advancements, several challenges remain, such as key knowledge gaps, algorithm selection, and bioinformatics software parametrization. In this mini-review, our primary focus is metagenomics, while recognizing that other -omics can enhance our understanding of the functional diversity of organisms and how they interact with the host. We aim to explore the current intersection of multi-omics, precision medicine, and machine learning in advancing our understanding of the gut microbiome. A multidisciplinary approach holds promise for improving patient outcomes in the era of precision medicine, as we unravel the intricate interactions between the microbiome and human health.

## Introduction

The human body hosts diverse communities of microbes and encompasses various glycomes, fostering diverse forms of communication with our organs and facilitating metabolic functions and molecular signals to maintain proper health (Kavanaugh et al., 2015; de Vos et al., 2022). The advent of high-throughput sequencing (HTS) along with research initiatives, such as the Human Microbiome Projects (iHMP Research Network Consortium, 2019), have paved a new path for microbial community characterization. The implementation of HTS strategies has equipped microbial taxonomic composition profiling at nearly any given body site, propelling the study of microbial networks, microbiome-disease associations, and host-microbiota interactions (Clooney et al., 2016). In parallel, the field of precision medicine has gained momentum, aiming to provide personalized healthcare solutions tailored to an individual’s unique genetic makeup, lifestyle, and environmental exposures (Schork, 2015). As we stand at the intersection of human microbiome research and healthcare innovation, there is a growing recognition that the gut microbiome and its exploration using -omics technologies hold immense potential as a key player in achieving the goals of precision medicine.

**FIGURE 1:**
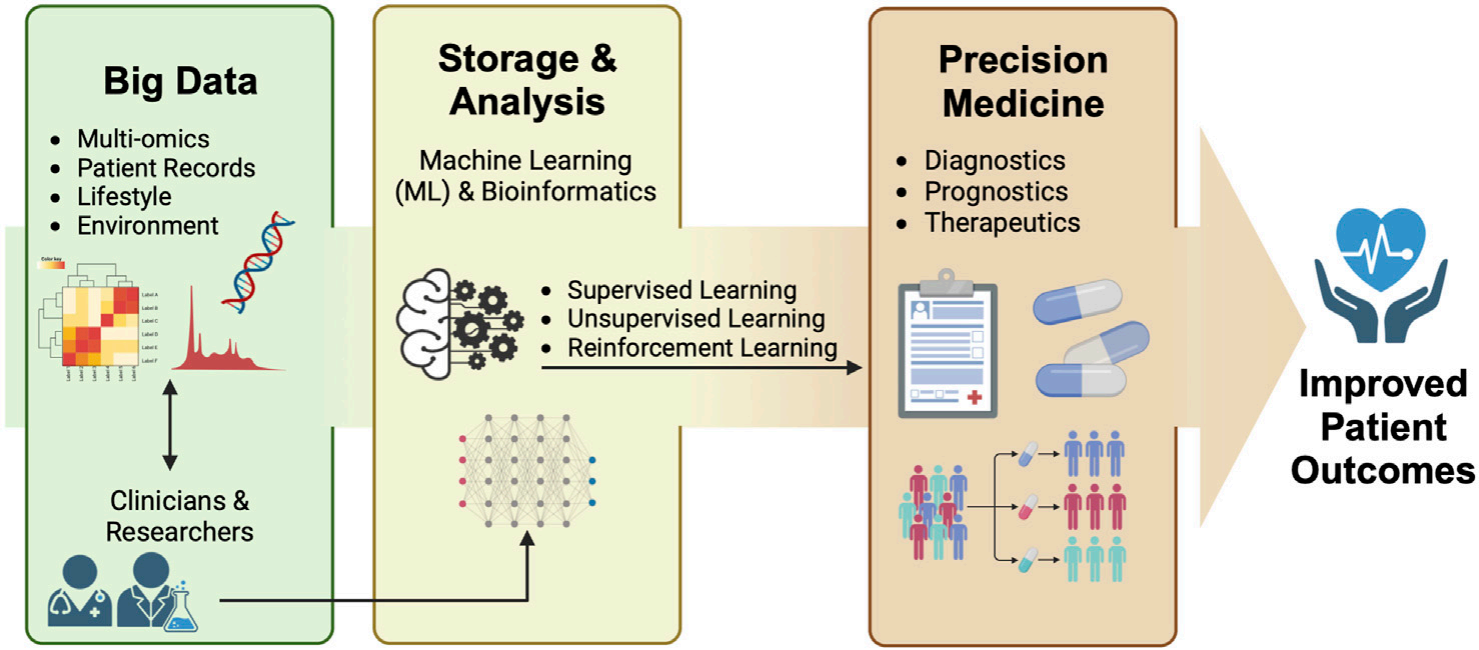
Researchers and clinicians harness the power of big data for downstream machine learning (ML) and bioinformatics analysis. This integrated approach yields valuable insights into the diagnosis, prognosis, and therapeutic treatment aspects of precision medicine, ultimately leading to improved patient outcomes.

Despite the prospect of the gut microbiome and ‘omics’ data used in support of precision medicine, the sheer complexity and large influx of these datasets pose formidable challenges to data interpretation and analysis. Hence, researchers have expanded their focus into the realms of bioinformatics and machine learning (ML) to address these challenges. This is done by utilizing the capacity of the aforementioned disciplines to integrate and process extensive data through different algorithms, enabling the development of models that can aid in diagnostic, prognostic, and therapeutic interventions. Harnessing these techniques enables the comprehensive analysis of intricate layers of biological information ranging from metagenomics to metabolomics, and the integration of patient record data, shedding light on the role of the gut microbiome in different aspects of precision medicine. This holistic approach ultimately improves the health trajectories of patients (Figure 1). This mini-review discusses the current status and interface between ML and bioinformatics methods of analyzing multi-omics data in advancing the understanding of the gut microbiome in relation to precision medicine.

## Methods for microbiome data analysis

The continuous evolution of genomic sequencing through multiple generations of sequencing technologies has resulted in our ability to determine the abundance of microorganisms at a granular level. To date, scientific research has primarily focused on characterizing bacterial species. However, clinical research has expanded to include other microorganisms in the gut, such as viruses, fungi, and helminths (Caporaso et al., 2012; Mukhopadhya et al., 2019; Berg et al., 2020; Rubel et al., 2020; Zhang et al., 2022). Microbiome investigation methods have expanded from 16S rRNA gene sequencing, whole-genome shotgun metagenomic sequencing, and RNA (metatranscriptomics) sequencing (Reuter et al., 2015). Here, we outline common sequencing platforms, methods, and databases used to investigate the gut microbiome.

### Sequencing technologies

Common second-generation sequencing (SGS) platforms and methods include Illumina, Ion Torrent, 454 Pyrosequencing, and SOLiD Sequencing (Liu et al., 2012; Meslier et al., 2022), where Illumina’s sequencing platform became more widely used for microbiome research due to their high-throughput processing, quality and consistency, cost-effectiveness, and relevant capabilities for microbiome research (Malla et al., 2018; Caporaso et al., 2012). SGS platforms generate short-read data, typically ranging from ∼50 to 300 base pairs in length and varying in sequencing depth (Ranjan et al., 2016; Johnson et al., 2019). Common third-generation sequencing (TGS) platforms include Helicos Single Molecule Sequencing (SMS), Pacific Biosciences (PacBio), and Oxford Nanopore Technologies (ONT) (Schadt et al., 2010; Koren and Phillippy, 2015; Athanasopoulou et al., 2021). SMS enables single-molecule detection without the need for amplification and was one of the early TGS technologies that had potential applications in studying complex microbial communities (Pushkarev et al., 2009). PacBio sequencing is a single-molecule, real-time (SMRT) sequencing technology that offers long-read sequence technology, where long-read data can range from 1,000–20,000 bases or more (Udaondo et al., 2021). This feature is beneficial for resolving complex microbial communities and detecting novel microorganisms. PacBio’s Sequel and Sequel II systems have been utilized in various metagenomic studies. ONT sequencing is based on nanopore technology, where DNA molecules pass through a nanopore, generating electrical signals that correspond to the nucleotide sequence. The MinION and PromethION devices from ONT have been used for real-time sequencing of microbial DNA, providing long-read lengths, portability, and potential for direct RNA sequencing. ONT sequencing also produces long-read data, where the read lengths can vary from several thousand base pairs to over a hundred thousand base pairs (Jain et al., 2016; Schmidt et al., 2017).

### Current paradigm: hybrid sequencing and bioinformatics

Combining short-read and long-read data has the capacity to characterize more complete and accurate microbial genomes present in the gut microbiome. Hence, a hybrid sequencing approach leveraging the strengths of both second and third-generation technologies has been increasingly used in gut microbiome research (Bharti and Grimm, 2021; Chen et al., 2022; Jin et al., 2022). To begin with, SGS is used to generate short-read sequences from gut microbiome samples. Whole genome short-read data is particularly useful for identifying the taxonomic composition of the microbiome, as short reads can accurately distinguish among microbial species. Long-read sequences from the same samples can then be generated, as they are crucial for resolving complex genomic regions, such as repetitive elements and structural variations, which are often missed or misassembled with short-read data (Mantere et al., 2019). Synergistically, short-read and long-read data can also be combined during the genome assembly process where long reads help anchor and scaffold the assembly, ensuring that the contigs represent more accurate and contiguous genome fragments. The short reads provide accurate base-level information to correct errors in the long reads and improve the overall accuracy of the assembly (Amarasinghe et al., 2020).

Study design plays a crucial role not only in selecting sequencing platforms or sequencing types but also in analyzing the vast amounts of data generated by various sequencing technologies in microbiome research. It is important to consider capturing extensive dietary, medical history, and patient lifestyle data in tandem with microbiome sampling and sequencing. This will allow a more comprehensive view of a disease state and potential patient outcomes, particularly when applying machine learning tools in a precise use case–as discussed later in the review. Relevant analyses include taxonomic profiling, phylogenetic analysis, metagenome or genome assembly, gene prediction, functional annotation, comparative metagenomics, pathway and differential abundance analysis, metatranscriptomics analysis, metabolomics, and network analysis (Prakash and Taylor, 2012; Maranga et al., 2023). Taxonomic profiling is one of the most important starting points for gut microbiome research; this is achieved using tools such as QIIME/QIIME2 (Quantitative Insights Into Microbial Ecology), mothur, Kraken/Kraken2 (Schloss et al., 2009; Caporaso et al., 2010; Wood and Salzberg, 2014; Bolyen et al., 2019; Wood et al., 2019; Lu and Salzberg, 2020; Schloss, 2020).

### Databases and algorithms

Numerous databases are essential for identifying organisms found within the gut microbiome and determining their taxonomic composition via diverse sequencing techniques. Commonly used repositories include NCBI’s non-redundant nucleotide database (NT), filtered NT, and Greengenes (McDonald et al., 2012; Shamsaddini et al., 2014). The NT is the most comprehensive sequence database and contains sequences from GenBank, RefSeq, PDB, and more, while the filtered NT is an expansion of RefSeq which contains organisms from NT whose phylogenetic lineage is clearly defined thus removing spurious and artificial sequences (Pruitt et al., 2005). While the former two databases consolidate information regarding an organism’s genes and genome, Greengenes leverages full-length 16S rRNA or metatranscriptomics data from public databases that fulfill several filtering qualifications (McDonald et al., 2012). The utilization of 16S rRNA presents a cost-effective approach, as it focuses on the identification of organisms through key marker genes originating from conserved genetic regions (Balvociute and Huson, 2017). Occasionally, this technique exhibits amplification bias to some extent and has limitations in the identification of archaea and viruses, as well as overall organism resolution, in contrast to metagenomics techniques such as random shotgun sequencing and WGS (Scholz et al., 2016).

Sequence aligners, especially established ones such as BLAST and Bowtie/Bowtie2, have been extensively reviewed (Langmead and Salzberg, 2012; Alser et al., 2021). Typically, BLAST is used for a handful of reads which is subsampled to search for similarities in a large database such as NT (Shamsaddini et al., 2014), while Bowtie and other similar rapid aligners are typically used to map reads to a set of reference genomes (Langmead, 2010). BLAST utilizes local alignment techniques to identify regions of similarity that have high-scoring pairs. This is exemplified by BLAST tools such as BLASTP, BLASTN, and BLASTX which function to identify similar pairs based on protein, nucleotide, and translated nucleotide sequences, respectively (Yang et al., 2014; Altschul et al., 1990; Chen et al., 2015; Li and Lu, 2019). This differs from Bowtie, which aligns millions of short reads from a sample of interest to limited number of references quickly. In light of this information, it is imperative for researchers to fully grasp the bioinformatics task at hand when deciding whether to employ BLAST or a short-read aligner.

## ML in gut microbiome for precision medicine: current research

Unlike traditional computer programming, machine learning (ML) algorithms are not explicitly programmed with rules and instructions. Instead, they autonomously learn patterns and relationships from data, allowing for generalization and predictions on new, unseen data (Bi et al., 2019). ML techniques are used in various applications to study the gut microbiome, such as microbiome composition, analysis, and therapeutic target identification (Fukui et al., 2020). There are three principal categories of ML: supervised, unsupervised, and reinforcement learning. Supervised learning, including linear regression, decision trees, and support vector machines, uses labeled datasets to train algorithms to classify or predict unknown outcomes. Unsupervised learning, including clustering, dimensionality reduction, and anomaly detection, cluster unlabeled datasets to discover hidden patterns or data groupings (Lopez et al., 2018; Cammarota et al., 2020). Reinforcement learning is a type of artificial intelligence (AI) that achieves a goal in an uncertain and potentially complex environment to build an ML model for decision-making by maximizing a reward function (McCoubrey et al., 2021). Several deep reinforcement learning models have been utilized for biomarker discovery as well as overall microbiome characterization (Mahmud et al., 2018; Liu et al., 2022b; Pan et al., 2022). The deployment of ML has the potential to catalyze novel advancements in patient risk assessment, the discovery of pivotal diagnostic biomarkers, and the prediction of treatment response outcomes.

### Personalized disease risk assessment

The unique variation in individuals’ gut microbiome profiles, much like a fingerprint, can be leveraged by recommended approaches, such as a patient-centered gut microbiome report, to aid clinicians in making personalized treatment decisions (King et al., 2019). ML can be leveraged in these instances to detect specific patterns in individual patients that can help identify early disease development. Early detection of cardiovascular disease, liver disease, endometriosis, and Type 1 and 2 diabetes mellitus are some of several diseases in which ML is being implemented to detect early disease onset (Aryal et al., 2020; Fernández-Edreira et al., 2021; Huang et al., 2021; Liu et al., 2022a; Ge et al., 2022). By recognizing the initial patterns of disease development, the possibility of reducing someone’s risk for a particular condition can be enhanced. This, in turn, allows for timely interventions and focused preventive measures that may impede or decelerate the disease’s advancement. This proactive strategy not only enhances the individual’s health outcomes but also alleviates the strain on healthcare systems and promotes overall public health.

### Diagnostic biomarker identification

Timely disease detection is vital, but not all individuals have routine access to health screenings for identifying symptoms at an initial stage. After the onset of a disease, the subsequent action may involve analyzing biomarkers to detect the presence of the condition. In these scenarios, ML can reveal essential biomarkers, enhancing the precision and effectiveness of disease detection and management (Uddin et al., 2019). Previous studies have implemented ML-based analysis to identify biomarkers associated with Graves’ disease, inflammatory bowel disease (IBD), and cancers such as colorectal cancer (CRC) (Zhou et al., 2018; Maurya et al., 2021; Zhu et al., 2021).

### Treatment response prediction

ML analysis of the human gut microbiome shows great potential in predicting response outcomes to interventions and medications (Ortega et al., 2014; de Jong et al., 2021). ML leverages metagenomic data to identify microbial patterns linked to treatment outcomes. These algorithms can then forecast how individual patients will react to interventions, such as dietary adjustments, probiotics, or medications, based on their unique gut microbiome profiles (Dahlin et al., 2022). This personalized approach enables healthcare providers to customize interventions for each patient, optimizing treatment effectiveness and minimizing adverse effects. Furthermore, ML can identify microbial biomarkers that indicate treatment success or failure, facilitating the development of more precise and efficient therapeutic strategies (Yi et al., 2021). As our understanding of the complex interactions between the gut microbiome and human health deepens, ML analysis becomes an invaluable asset in advancing precision medicine and enhancing patient-targeted outcomes.

## Clinical applications of gut microbiome data

### Diagnosis and prognosis

A variety of multi-omics approaches, including microbial metabolic modeling and phenome-wide associations, are used to identify metabolites and other biomarkers associated with distinct irritable bowel syndrome (IBS) subtypes, IBD, necrotizing enterocolitis, and late-onset sepsis (Stewart et al., 2016; Stewart et al., 2017; Grasberger et al., 2021; Jacobs et al., 2023). These findings contribute to the translation of research discoveries into clinical applications, bridging the gap between laboratory research and improved patient care. One ML approach, trans-omic network analysis, has successfully identified patterns in blood parameters, gut microbiome, and urine metabolome data to identify biomarkers associated with carotid atherosclerosis (Li et al., 2021). Additionally, gene-microbiome association methods employed multi-omics techniques of transcriptomic and metagenomic profiling to expand clinical understanding of the pathophysiology of CRC, IBD, and IBS (Priya et al., 2022).

The gut microbiome’s impact on the body as a whole system is evident in its ability to influence various disease stages, with neurological and cancer-related conditions being particularly susceptible to its effects (Aho et al., 2021; Dizman et al., 2022; Tian et al., 2022). Recent advances in gut microbiome and multi-omics data allow for an augmented understanding of disease and symptom severity, disease progression, and predicted responses to therapeutic treatments. Longitudinal multi-omics was used to associate the severity of IBS symptoms with changes in bacterial relative abundance, and specific bacterial species were found to be associated with “flares” in patient symptoms (Mars et al., 2020). Favorable and unfavorable responses to IBS therapeutic classes were identified using high-throughput -omic profiling and the predictive accuracy improved significantly with the incorporation of proteomics, metabolomics, and metagenomics data (Lee et al., 2021). Microbiota compositions of patients with ulcerative colitis were used to identify proteases associated with disease severity (Mills et al., 2022). In hematopoietic cell transplant patients, microbial diversity was used to predict critical outcomes (Adhi et al., 2019).

### Therapeutic treatment

Common gut microbiome-based interventions are additive and modulatory therapies. Additive therapies, as the name suggests, involve introducing microorganisms to a patient’s gut microbiome. Fecal Microbiota Transplantation (FMT) and probiotics are effective additive therapies. FMT, also known as fecal bacteriotherapy, involves the introduction of fecal matter into a patient’s gut microbiome and has been found to improve patient outcomes related to *Clostridium difficile* infection (CDI), hepatic encephalopathy, and blood disorders with antibiotic-resistant bacteria (Petrof et al., 2013; Bilinski et al., 2017; Zuo et al., 2018; Bajaj et al., 2019). Studies involving probiotic therapies have shown improvements regarding obesity-related disorders and cirrhosis (Dhiman et al., 2014; Depommier et al., 2019). Live biotherapeutic properties have recently been approved by the Federal Drug Administration (U.S. Food and Drug Administration, 2023) and have been an effective treatment for IBS and recurrent CDI (Khanna et al., 2021; Khanna et al., 2022; Quigley et al., 2023). Another well-known type of therapeutic treatments are modulatory ones: diet and exercise. The introduction or restriction of certain nutrients is known to affect gut microbiome composition and improve nonalcoholic fatty liver disease and cardiovascular disease outcomes (Levitan et al., 2009; Lopez-Garcia et al., 2014; Mardinoglu et al., 2018).

## Concluding remarks

The development of ML, sequencing technologies, and bioinformatics pipelines have enabled the use of the gut microbiome knowledge to improve the health outcomes of patients. However, it is important to acknowledge the current limitations in this field. A myriad of sequencing and bioinformatics combinations can be selected for the application and translation of microbiome analysis in precision medicine that can lead to widely varying results. Consequently, a principled approach should be applied based on the study design and each step of the process should be determined to ensure high-quality data collection and thoughtful algorithm selection while aiming to document all steps using technologies such as BioCompute Objects (Simonyan et al., 2017). This would allow better interpretation of results by clinicians and other researchers.

The prospect of personalized healthcare is becoming more and more tangible as our understanding of this field deepens. While there are software packages and toolkits available for multi-omics research devoted to the clinical understanding of disease, the output from the software often lacks user-friendly reports for clinicians. Running these tools typically demands a high level of technical expertise, which is essential to maintain the validity of the results. Future endeavors in multi-omics and machine learning would be best served with a multidisciplinary approach, to develop reporting mechanisms of the results that allow evidence based clinical decision-making. These efforts have the potential to harness the full capabilities of multi-omics approaches in elucidating the gut microbiome and further advancing precision medicine. Addressing these limitations will be crucial to translate this vision into reality and benefit an extensive community.
